# Unveiling Swift Heavy Ion Track Morphology in Sr-Based High-Entropy Perovskites

**DOI:** 10.1021/acsnano.5c13654

**Published:** 2026-01-10

**Authors:** Ashish Kumar Gupta, Eva Zarkadoula, Brianna L. Musico, Jordan A. Hachtel, Manuel A. Roldan, Vikas Reddy Paduri, Colby Harris, Ramji Subedi, Maxim Ziatdinov, Sergei V. Kalinin, Christina Trautmann, Veerle Keppens, Jie Liu, Yanwen Zhang, William J. Weber, Ritesh Sachan

**Affiliations:** 1 School of Mechanical and Aerospace Engineering, 7618 Oklahoma State University, Stillwater, Oklahoma 74078, United States; 2 Center for Nanophase Materials Sciences, 6146 Oak Ridge National Laboratory, Oak Ridge, Tennessee 37831, United States; 3 Sigma Division, 5112 Los Alamos National Laboratory, Los Alamos, New Mexico 87545, United States; 4 Eyring Materials Center, 630705 Arizona State University, Tempe, Arizona 85287, United States; 5 Physical Sciences Division, Pacific Northwest National Laboratory, Richland, Washington 99352, United States; 6 Department of Materials Science and Engineering, 122608 University of Tennessee, Knoxville, Tennessee 37996, United States; 7 29090 GSI Helmholtzzentrum für Schwerionenforschung, Darmstadt 64291, Germany; 8 Institute of Modern Physics, 71200 Chinese Academy of Sciences, Lanzhou 730000, China; 9 Department of Mechanical and Materials Engineering, Queen’s University, Kingston, Ontario K7L3N6, Canada

**Keywords:** high-entropy oxides, high-entropy perovskite, swift heavy ion irradiation, electron irradiation, phase transformation, data science in microscopy

## Abstract

The incorporation of multiple cations on a single lattice site in the high-entropy oxides is considered the key driving factor for modifying the known atomic-level response to energetic ion irradiation due to the presence of structural disorder; however, these effects are not well-understood yet. In this work, we present atomic-level insight into irradiation-induced nanoscale phase transformations in a perovskite-structured high-entropy oxide, Sr­(Zr_0.2_Sn_0.2_Ti_0.2_Hf_0.2_Nb_0.2_)­O_3_ (Sr­(HE)­O_3_), subjected to 774 MeV swift Xe heavy ions, where damage is dominated by inelastic ion–lattice interactions. While these ions generally are known to create nanoscale disordered channels, “ion tracks”, along the penetration direction in the material, this study shows the formation of discontinuous and partially recrystallized ion tracks in Sr­(HE)­O_3_. Compared to SrTiO_3_ irradiated under identical energy loss conditions, the ion tracks in Sr­(HE)­O_3_ exhibit significantly reduced diameters and a markedly different interfacial structure. Notably, the crystalline–amorphous interface in Sr­(HE)­O_3_ shows minimal lattice distortion, confined to approximately 2–3 monolayers, in contrast to the extended disordered shell commonly observed in SrTiO_3_. Using in situ atomic-resolution electron microscopy, we further demonstrate that the amorphous/disordered regions within Sr­(HE)­O_3_ ion tracks remain highly stable under electron irradiation, whereas tracks in SrTiO_3_ readily recrystallize. This enhanced stability is attributed to the dominance of structural and chemical complexity arising from multiple B-site cations, which suppress defect migration and templated recrystallization driven by electronic excitations and local heating. Overall, this study highlights how high-entropy oxide chemistry fundamentally reshapes irradiation damage evolution, offering insights into defect formation and phase stability under extreme conditions.

## Graphical abstract

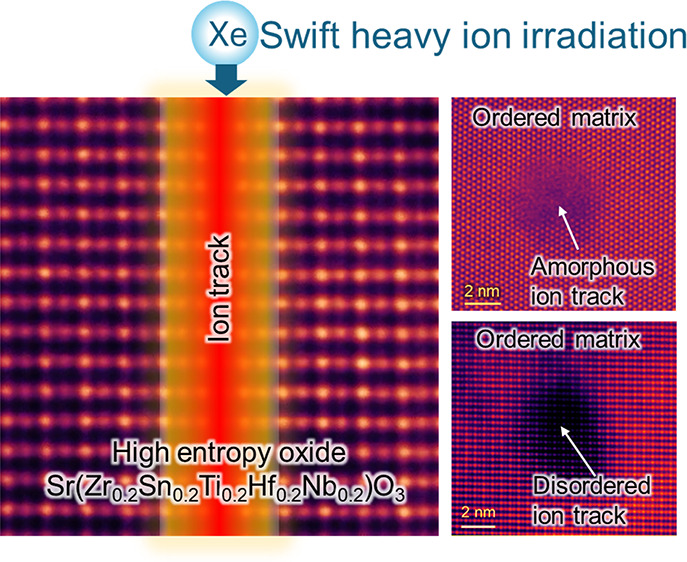

Understanding the response of ceramics under ion irradiation has always enthralled the research community due to its ability to modify these materials, leading to unknown properties and functionalities. Ion irradiation effects on ceramics, particularly metal oxides, have been investigated extensively owing to their evident impact on developing radiation-tolerant materials for nuclear, high-energy accelerators and space applications, as well as the broader impact in, e.g., designing microelectronics and catalytic materials and enabling new phases with fast ionic transport.^1–5^ Progress in understanding ion–solid interactions has revealed insight into defect structures, interfacial strain, and metastable phase formation in oxides, which has, in turn, enabled the design of functional materials and cutting-edge device technologies.^6–9^ However, the constant need to search for advanced materials that exhibit outstanding properties or significantly improve existing ones has been a motivation behind shifting the attention from traditional metal oxides toward new candidates that leverage local chemical disorder and atomic bonding engineering. The recent emergence of high-entropy oxides (HEOs) that consist of multiple cations (typically five or more) on an ordered lattice site has offered one such alternative due to its their versatile elemental combinations and resulting tunable physical properties as compared to conventional oxides.^10^ These HEOs fit very well in the scope of this research shift, where ion irradiation is expected to either facilitate the order/disorder transformation leading to metastable phases or demonstrate high radiation tolerance against defect formation from energetic ions.^11–13^

However, the grand challenge is to develop a fundamental understanding of the defect formation and phase transformation in HEOs upon ion irradiation and draw scientific conclusions on the materials response, which are still highly limited to date with very few reported studies.^14,15^ Although these studies are conducted on HEOs, as well as other multicomponent ceramic systems (diborides,^16,17^ carbides,^18^ and nitrides^19^), a common attribution for a benign or significant change in the response to irradiation is given to the structural disorder created in the high-entropy or compositionally complex systems due to the incorporation of multiple cations, as compared to the simple metal oxides. While this phenomenon appears to be similar to the irradiation response of metallic high-entropy alloys, HEOs exhibit more complex scenarios due to the presence of multiple cationic and anionic lattice sites. The reported studies include investigations on the radiation stability and mechanical properties of various HE pyrochlores, such as (La_0.2_Ce_0.2_Nd_0.2_Sm_0.2_Gd_0.2_)_2_Zr_2_O_7_ irradiated with 9 MeV Au ions,^20^ (Yb_0.2_Tm_0.2_Lu_0.2_Ho_0.2_Er_0.2_)_2_Ti_2_O_7_ irradiated with 4 MeV Au ions^11^ and with 600 keV Xe ions,^21^ A_2_Ti_2_O_7_ (A = Gd, Dy, Ho, Er, Yb, and Nd)^22^ irradiated with 3 MeV Au ions, (Sm_0.2_Eu_0.2_Gd_0.2_Dy_0.2_Er_0.2_)_2_Hf_2_O_7_ irradiated with 400 keV Kr ions,^23^ and compositions with varying both A- and B-site cations.^24^ Among these studies, some have shown a modest improvement in radiation tolerance for the knock-on damage attributed to the large lattice distortion,^11,20^ whereas another study indicates that (La_0.2_Nd_0.2_Sm_0.2_Eu_0.2_Gd_0.2_)_2_Zr_2_O_7_ and Gd_2_(Ti_0.25_Zr_0.25_Hf_0.25_Ce_0.25_)_2_O_7_ hold promise of enhanced radiation resistance across many multicomponent pyrochlore ceramics under irradiation of 800 keV Kr ions.^24^ While these studies clearly focus on the elastic energy transfer (nuclear energy loss) from the irradiated ions, the understanding of inelastic interactions (electronic energy loss) in HEOs caused by ionizing swift heavy ion (SHI) irradiation is simply unknown and is the primary emphasis of the present work. It is also worth emphasizing that much of the work in the literature on HEO response to irradiation has been focused on pyrochlore structures and this work is expanding it to ABO_3_-type perovskite oxides, which have demonstrated a high impact as functional materials.

It has been demonstrated that the SHI irradiation of metal oxides is predominantly governed by the ionization and electronic excitation processes. A significant part of the electronic energy loss of each ion is transferred to the lattice via electron–phonon coupling. The ultrafast energy transfer to the atoms can cause local melting along the ion trajectory (thermal spike), followed by quenching and in some materials partial recrystallization. The remaining frozen-in damage zone is denoted as the ion track. Tracks have a cylindrical shape and a diameter of a few nanometers. Depending on the material, tracks can be amorphous or, in some materials, consist of a disordered crystalline core surrounded by a defect-rich halo. The characteristics of ion tracks vary primarily due to the variation in thermal conductivity and electron–phonon coupling in different material systems. These effects are reflected in the form of (i) differences in the track diameter,^25–27^ (ii) presence of amorphous to disordered phases^28–30^ or core–shell phase morphology,^31–33^ and (iii) a continuous or discontinuous damaged morphology along the length of the ion tracks.^34,35^ In the case of perovskite oxides (e.g., SrTiO_3_), which are considered as the building block for various functional applications, defect evolution and phase formation via irradiation methods,^36–38^ including SHIs, have demonstrated that atomic rearrangement enables novel electronic, magnetic, and optical properties and thus requires an advanced atomic-level structural understanding. The study of irradiation-induced effects on the atomic structure is even more critical due to the presence of multiple cations at one or more lattice sites in HEOs, which can pave the pathway to processing directions from both the fundamental viewpoint and applications.

In this work, we present atomic-level insights into the ion tracks formed in high-entropy Sr­(Zr_0.2_Sn_0.2_Ti_0.2_Hf_0.2_Nb_0.2_)­O_3_ (abbreviated as Sr­(HE)­O_3_) by 774 MeV swift heavy Xe ions that have a dominating electronic energy loss regime extending up to ∼20 μm. For the proposed Sr­(HE)­O_3_, the Goldschmidt tolerance factor (*t*), which indicates the degree of distortion within the material, is estimated using the relationship *t* = (*r*_a_ + *r*_o_)/√2­(*r*_b_ + *r*_o_), where *r*_A_, *r*_B_, and *r*_O_ are the radii of the A cation, B cation, and oxygen anion, respectively. Based on the Shannon ionic radii, the tolerance factor is calculated to be 0.969 and suggests that Sr­(HE)­O_3_ stabilizes as a cubic perovskite structure, making it structurally comparable with well-known cubic SrTiO_3_.^39,40^ The ion track morphology in Sr­(HE)­O_3_ is compared with that formed in SrTiO_3_ with similar electronic energy losses to understand the role of multiple cations. Subnanometer-level studies of atomic arrangement and lattice distortions are conducted by atomic-resolution imaging using scanning transmission electron microscopy (STEM) and data-driven image analysis. Since the ion tracks in SrTiO_3_ readily recrystallize under prolonged electron beam exposure,^32,41^ we further study the effect of electron irradiation and phase stability of tracks in Sr­(HE)­O_3_ via in situ STEM observations. Material properties relevant to track creation within the inelastic thermal spike (iTS) model are further discussed.

## Results and Discussion

To investigate how multiple B-site cations in Sr­(HE)­O_3_ affect the ion track morphology, Sr­(HE)­O_3_ and SrTiO_3_ samples irradiated under very similar conditions were both STEM-analyzed near the surface at a depth of ∼5 μm, as illustrated in Figure 1. SRIM simulations confirm that the electronic energy loss remains constant at ∼28.5 keV/nm up to depths of ∼18 μm in Sr­(HE)­O_3_ and ∼10 μm in SrTiO_3_, after which it declines sharply (Figure 1a). Although the electronic energy loss profiles diverge at greater depths, the ∼5 μm region was selected to ensure a controlled comparison of the ion track morphology under similar energy loss conditions.

**Figure 1:**
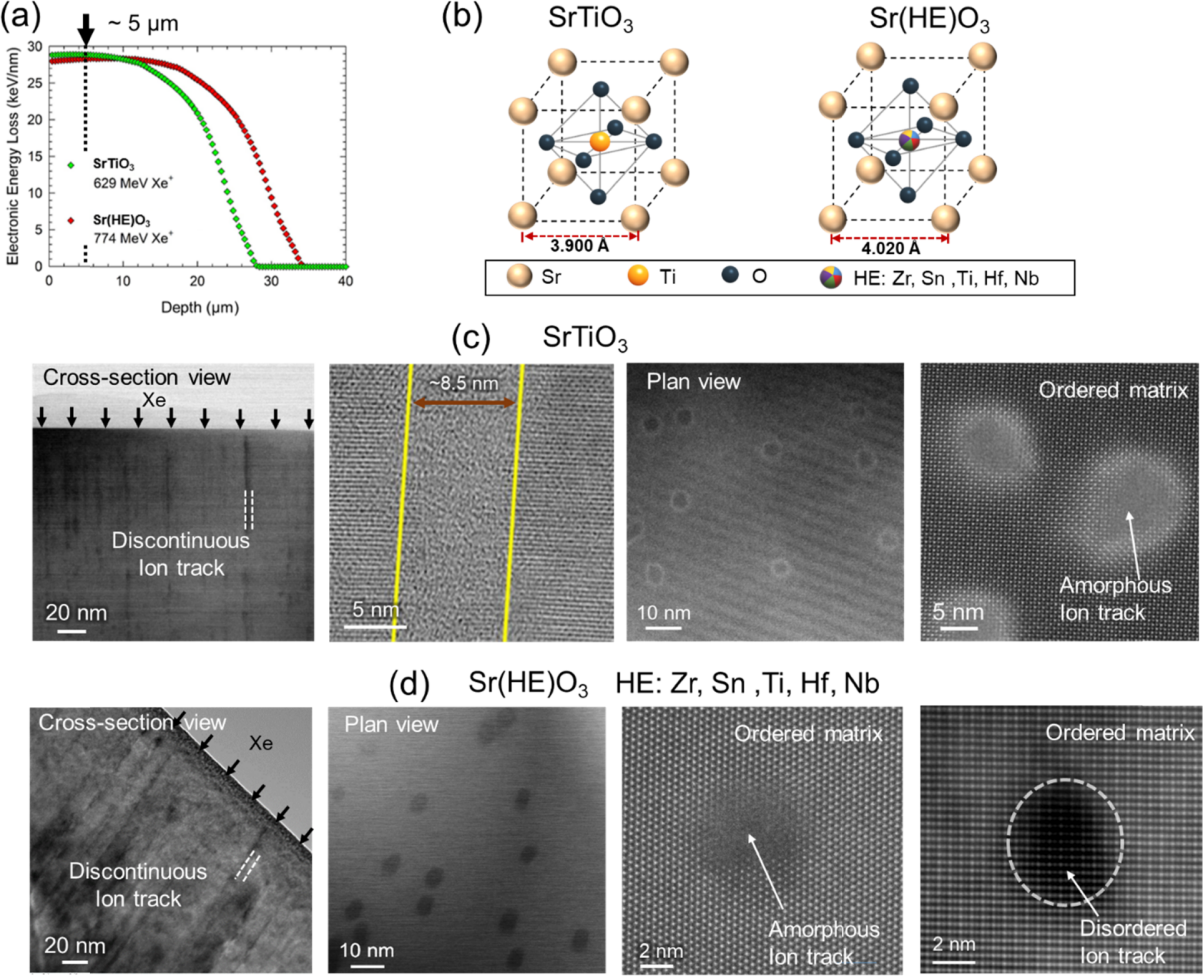
(a) Electronic energy loss as a function of penetration depth in Sr­(HE)­O_3_ and SrTiO_3_ for 774 and 629 MeV Xe ion irradiation, respectively; (b) atomic structure models of Sr­(HE)­O_3_ and SrTiO_3_; cross-sectional and plan-view images of formed ion tracks in (c) SrTiO_3_ and (d) Sr­(HE)­O_3_. The cross-sectional images in both SrTiO_3_ and Sr­(HE)­O_3_ show the formation of discontinuous tracks. A magnified cross-sectional image of the continuous portion of a track in SrTiO_3_ is shown to demonstrate the track diameter. Plan-view images of tracks in SrTiO_3_ show an amorphous core with a disordered (brighter contrast) shell in the ordered matrix. On the contrary, Sr­(HE)­O_3_ consists of ion tracks with two different morphologies: (i) completely amorphous and (ii) disordered crystalline ion tracks as shown in (d) (right two images).

From a structural perspective, Sr­(HE)­O_3_ comprises multiple B-site cations (Zr, Sn, Ti, Hf, and Nb), forming a chemically disordered perovskite matrix, in contrast to the single B-site cation (Ti) in the ordered SrTiO_3_ perovskite (Figure 1b). Cross-sectional and plan-view TEM images reveal that ion irradiation induces discontinuous ion tracks in both materials (Figure 1c,d), albeit with markedly different characteristics. In SrTiO_3_, these discontinuous tracks have previously been reported and attributed to partial lattice melting driven by energy deposition near the critical energy loss threshold required for track formation. Discontinuous tracks can also arise from enhanced defect recombination and thermal diffusion, both of which are characteristic of perovskite structures.

A key observation is the significant reduction in ion track diameter in Sr­(HE)­O_3_ (∼5 ± 2 nm) compared to SrTiO_3_ (∼8.5 ± 1 nm), although created under identical energy loss conditions. In SrTiO_3_, tracks exhibit a core–shell structure with distinct peripheral contrast, indicating a strained, disordered but crystalline shell. In contrast, ion tracks in Sr­(HE)­O_3_ show minimal or no lattice distortion at the interface between the amorphous region and the surrounding crystalline matrix. More importantly, regardless of the grain orientation, we observe two distinct morphologies that are consistently observed in Sr­(HE)­O_3_, (i) completely amorphous and (ii) disordered crystalline ion tracks, suggesting that these structural states emerge from the dynamic processes of track discontinuity and local recrystallization during the cooling of the thermal spike. In the following, we further analyze the atomic order/disorder in and around these tracks.

The two heat diffusion equations (eqs 1 and 2) of the inelastic thermal spike model calculated the radial energy deposition due to 629 and 774 MeV Xe ions in SrTiO_3_ and Sr­(HE)­O_3_, respectively (Figure 2). As expected, the smaller thermal conductivity of Sr­(HE)_3_, its larger e-ph mean free path, and smaller specific heat capacity result in slightly smaller energy deposition several nanometers around the ion path compared to SrTiO_3_. This trend is in good agreement with the smaller track size observed in Sr­(HE)­O_3_. Compared to SrTiO_3_, the B-site cation in Sr­(HE)­O_3_ is partially substituted with larger atoms (Zr, Hf, and Nb are larger than Ti, and only Sn is smaller in size). This size mismatch hinders diffusion across the B sublattice. In addition, the fluctuations in the local B–O bond length due to the presence of atoms of multiple elements at the B-site lead to high strain energy locally, which thus inhibits atomic diffusion. The local tolerance factor due to different B-site cationic radii is calculated to be ∼0.043 with a finite spread of ∼0.02, which also is reflected by ∼2.1% variation in B–O bond length. The reduced atomic mobility and diffusion make recrystallization more difficult in Sr­(HE)­O_3_.

**Figure 2:**
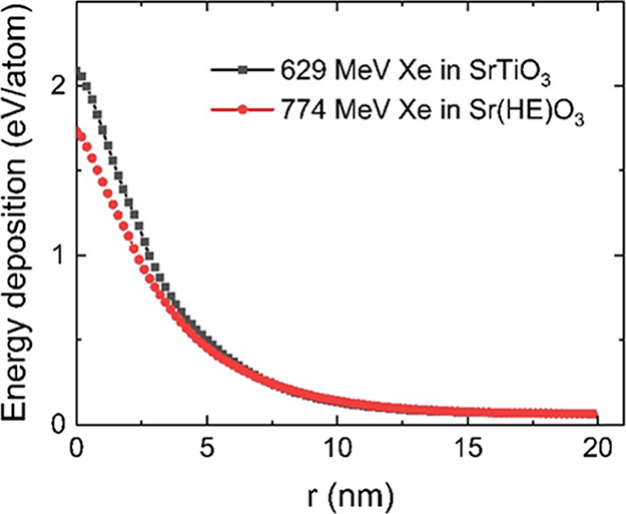
Energy deposition profiles as a function of the distance *r* from the ion path due to 629 and 774 MeV Xe ion irradiation in SrTiO_3_ and Sr­(HE)­O_3_, respectively, calculated with the iTS model.

Given that ion track formation involves ultrafast melting followed by rapid quenching on the timescale of ∼100 ps, the possibility of localized compositional changes within the track region has been examined using STEM/EDS, as presented in Figure 3. Elemental maps of the A-site cation (Sr) and B-site cations (Zr, Ti, Nb, Sn, and Hf) have been acquired across regions encompassing both the ion track and the surrounding ordered matrix. These compositional maps reveal a homogeneous distribution of all elements with no discernible variation between the ion track and the surrounding matrix. This indicates that ion track formation in Sr­(HE)­O_3_ does not induce any significant compositional redistribution. Similar findings have been reported in other radiation-tolerant oxides, such as the pyrochlore-structured Gd_2_Ti_2_O_7_, where the compositional uniformity was largely preserved despite localized amorphization. The absence of significant elemental segregation in Sr­(HE)­O_3_ can be attributed to two key factors: (i) the ultrafast nature of the thermal spike associated with SHI irradiation, which limits the time available for long-range atomic migration, and (ii) the sluggish atomic diffusion inherent to high-entropy systems. Variations in the elemental composition ranging within 0–10 atom %, however, are observed at the ion track center, specifically for Sr. While these variations occur primarily due to the stochastic nature of ion track formation where recrystallization completes with the rapid solidification, they should not be specific to Sr ions. These fluctuations could also be attributed to the local short-range order in HEOs. Recent works on HEOs, including spinel, pyrochlore, perovskite, and rock-salt structures, demonstrated subangstrom structural and chemical changes due to such local short-range order.^42,43^ Interestingly, the overall composition of oxygen anions is also observed to be unchanged within the measurement accuracy. However, it is reasonable to speculate that disordering of the oxygen anions occurs during this process. This hypothesis is further investigated using EELS analyses of ion tracks as shown in Figure 4.

**Figure 3:**
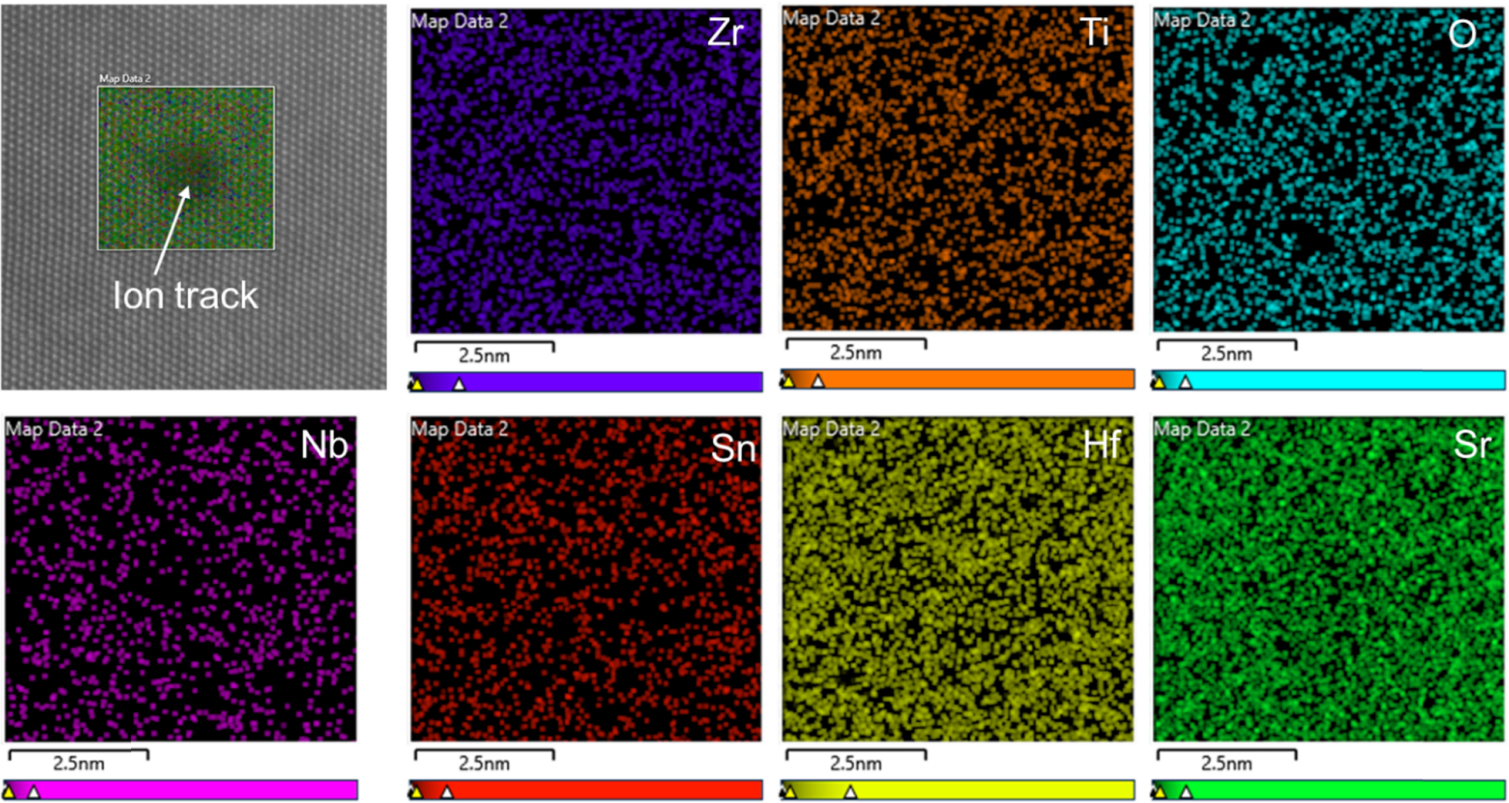
HAADF image of a single ion track (top left) in Sr­(HE)­O_3_. The white frame indicates the analysis box of EDS elemental mappings of various elements. No significant compositional change in or around the ion track is observed.

**Figure 4:**
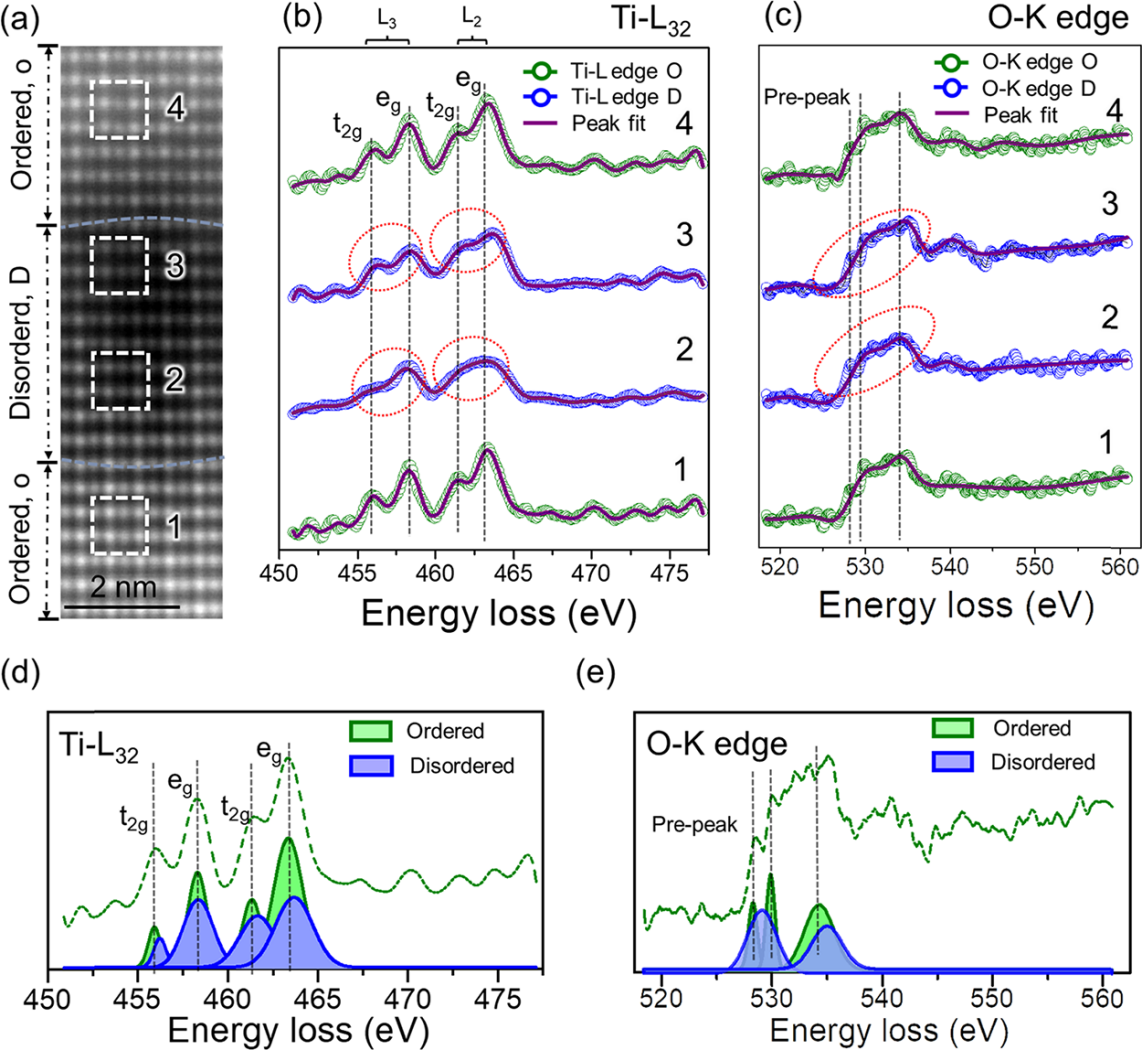
(a) ADF image of a region of Sr­(HE)­O_3_ containing an ion track (disordered 2 and 3) with the ordered matrix below (1) and above (4) the track. Energy loss spectra of (b) Ti L_32_ edge (456 eV) and (c) O K edge (532 eV) spectra for the region imaged in (a). (d, e) EELS spectra representative of ordered and disordered areas with Gaussian fits showing the changes in the peak position and broadening with phase transformation.

In the ADF image (Figure 4a), regions 1 and 4 correspond to the ordered matrix, while regions 2 and 3 are located within the disordered area of the ion track. Among the constituent B-site elements on the high-entropy sublattice, i.e., Nb, Hf, Zr, Ti, and Sn, only Ti exhibits a well-defined core-loss absorption edge with a distinct fine structure (Ti L_32_ at ∼455 eV). The other elements either show core-loss edges at significantly higher energy losses or lack sufficient spectral resolution for probing local bonding environments.^42^ Therefore, the analysis focuses on the Ti L_32_ and O K (∼532 eV) absorption edges, which are particularly sensitive to changes in local atomic coordination. The corresponding EELS spectra for the Ti L_32_ and O K edges are shown in Figures 4b and 4c, respectively. The Ti L_32_ edge arises from spin–orbit splitting of Ti 2p orbitals (2p_3/2_ and 2p_1/2_) into transitions to unoccupied 3d states, and the O K edge results from O 1s to 2p transitions, both of which are widely used to probe cation coordination and defect chemistry.^43,44^ In the ordered regions (1 and 4 in Figure 4a), the Ti L_2,3_ spectrum displays four distinct peaks, two each in the L_3_ and L_2_ edges, corresponding to crystal field splitting of the 3-fold t_2g_ (d_*xy*_, d*yz*, and d_*xz*_ orbitals) and 2-fold e_g_ ( $3d_{z^{2}}\mathrm{and}3d_{x^{2}‐y^{2}}$ orbitals) states.^44^ This splitting, observed in perovskite oxides such as SrTiO_3_, is highly sensitive to the local symmetry and bonding environments. Comparison of the disordered regions (2 and 3) with the ordered ones (1 and 4) reveals two key observations: (i) Crystal field splitting still exists in the disordered phase (regions 2 and 3 in Figure 4b) of the ion track despite the significant change in the atomic structure, suggesting the presence of short-range ordering in Ti–O octahedra. (ii) The e_g_ and t_2g_ peaks of the L_32_ edge show a position shift and broadening indicating the distortions in the Ti–O octahedra and transitioning to polyhedra.^30,45^ The spectra fitted with various peaks of Gaussian fit models clearly demonstrate these changes in Figure 4d. During a transition from an ordered to disordered lattice under irradiation, the local Ti–O coordination can become distorted, reducing the crystal field strength and symmetry around Ti. This results in a slight shift of the Ti L_3__2_ edge toward lower energy and broadening or diminished splitting of the t_2g_–e_g_ peaks, indicative of a partial reduction of Ti^4^^+^ to Ti^3^^+^ or an increase in oxygen vacancy concentration.^46,47^ Such effects have been reported in defect-rich or disordered SrTiO_3_ regions, where local distortions disrupt the long-range periodic potential, weakening the local electron structure and the ligand field. Similar trends are observed on the O K edge. In the ordered regions, a well-defined prepeak, typically attributed to hybridization between O 2p and Ti 3d states, becomes broadened and merges with the main peak in the disordered ion track, reflecting the breakdown of the long-range order in oxygen coordination.^30^

To investigate the atomic arrangement in and around ion tracks in Sr­(HE)­O_3_, detailed atomic-scale analysis has been performed using computational image processing of atomic-resolution ADF-STEM images, as shown in Figure 5. Atomic-resolution ADF images have been acquired for multiple ion tracks exhibiting (i) disordered crystalline and (ii) fully amorphous morphologies. For both track categories, atomic column positions were extracted based on *Z*-contrast intensity. In the cation mapping along the [011] zone axis, Sr atoms at the A-site (yellow) and high-entropy elements at the B-site (pink) were distinctly identified. Figures 5c and 5f display the atomic column maps corresponding to the disordered crystalline (Figure 5b) and amorphous (Figure 5d) tracks in the ordered matrix, respectively. In the disordered crystalline tracks (Figure 5b), no significant displacement of Sr or HE cation columns is observed relative to the surrounding ordered lattice, indicating that the cationic sublattice remains largely preserved. However, pronounced variations in background contrast and atomic column intensities suggest substantial disruption within the oxygen sublattice. In contrast, amorphous ion tracks exhibit a sharp interface with the crystalline matrix, with disorder confined to approximately 2–3 monolayers at the boundary, which is identified based on the atomic intensities (Figure 5d). Figure 5g presents a quantitative comparison of Sr and HE column intensities at the disordered interface versus those at the adjacent ordered matrix, revealing a consistent reduction in intensity across the interfacial region. This indicates that both A-site and B-site cations experience some level of intensity modulation, while their lattice positions remain largely intact. The present findings demonstrate the preservation of the lattice arrangement at subnanometer (angstrom-level) resolution, which is significant for understanding radiation-induced effects that may evolve with directional lattice strain. Nevertheless, fluctuations in lattice distortion and local chemical composition have been reported in high-entropy oxides (HEOs) due to short-range ordering among multiple B-site cations at the subangstrom scale.^42,43^ Such fluctuations are inherently random, reflecting the intrinsic characteristics of HEOs. The distinct intensity variations observed in Figure 5g, along with their distribution spread, are consistent with previously reported results and become more apparent as the intensities shift from ordered to disordered regions. Overall, this observation clearly highlights the structural stability of the cationic framework even under intense ionizing irradiation.

**Figure 5:**
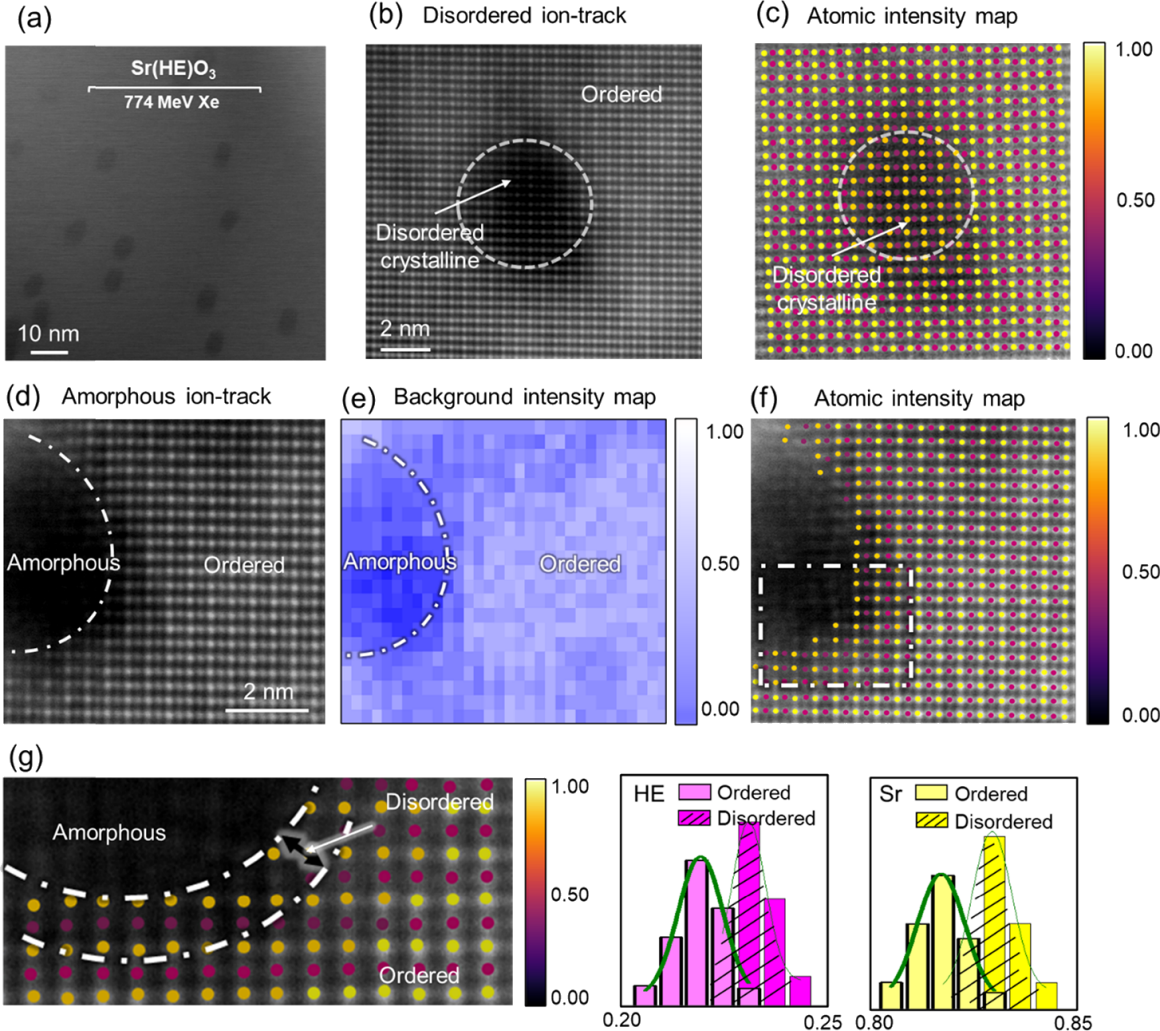
(a) Plan-view ADF image showing ion tracks in a Sr­(HE)­O_3_ sample. (b) Atomic-resolution ADF image of a single disordered, crystalline ion track with the corresponding (c) atomic intensity map. (d) Atomic-resolution ADF image of a single amorphous ion track with the corresponding (e) background and (f, g) atomic intensity maps. (e) Distribution of Sr and HE cation intensities in ordered and disordered crystalline regions.

The behavior of Sr­(HE)­O_3_ parallels the observations in pyrochlore oxides (A_2_B_2_O_7_), where amorphous ion tracks are typically surrounded by narrow (∼2–3 monolayers) disordered regions.^4,5,30,34^ For example, in SHI-irradiated Gd_2_Ti_2_O_7_, a peripheral defect-fluorite zone around the ion track is stabilized by strain-assisted oxygen migration during the rapid melt-quench event. Compositional complexity at the B-site, as in Gd_2_TiZrO_7_ ^28,31^ or Y_2_Sn_2__–__*x*_Zr_*x*_O_7_,^48^ has been reported to promote phase transformation and increase the extent of disordered regions. In comparison, Sr­(HE)­O_3_ exhibits a more spatially confined and sharply defined disordered phase, underscoring the irradiation resistance of its cationic lattice and the mitigating influence of high compositional entropy on the track morphology.

On another note, while the contrast in ADF images of amorphous ion tracks is clearly distinguished between the amorphous center, the peripheral disordered zone, and the ordered matrix region, the variation in the intensities of atomic columns in disordered crystalline ion tracks can be attributed to either (i) the formation of oxygen-deficient disordered phases during ultrafast solidification or (ii) projection overlap of amorphous and recrystallized segments within discontinuous tracks. In amorphous ion tracks, the contrast variation at the periphery (disordered phase) is notably weaker, especially for features associated with anionic disorder. This likely points out that the disordered phase morphology of ion tracks appears due to the overlapping contributions from amorphous and partially recrystallized regions projected along the track depth, resulting in more diffuse contrast signatures. However, the possibility of overlapping disordered regions cannot be entirely ruled out, as disorder in the oxygen sublattice may occur during recrystallization, leading to mixed atomic column intensities.

To compare ion track morphologies, atomic-scale analyses were performed on ion tracks formed in SrTiO_3_, as shown in Figure 6a–f. As previously mentioned, the average ion track diameter in SrTiO_3_ was measured to be ∼8.5 ± 1 nm, notably larger than the ∼5 ± 2 nm observed in Sr­(HE)­O_3_. The ion tracks in SrTiO_3_ exhibit an amorphous core region surrounded by a strained disordered shell. These distinct zones can be differentiated based on contrast variations in ADF images, with the disordered regions displaying enhanced background intensity due to strain fields induced by irradiation-generated defects.^32,36^ Mapping of background intensity, which serves as a proxy for the order/disorder in the O sublattice, reveals an increase in intensity in the peripheral disordered zone, consistent with distortions of TiO_6_ octahedra.^32,49,50^ Such behavior aligns with previous reports on laser-irradiated SrTiO_3_ exposed to a single pulse of a nanosecond laser, where subsurface disordered layers associated with Ti–O lattice distortions were observed.^36,37^

**Figure 6:**
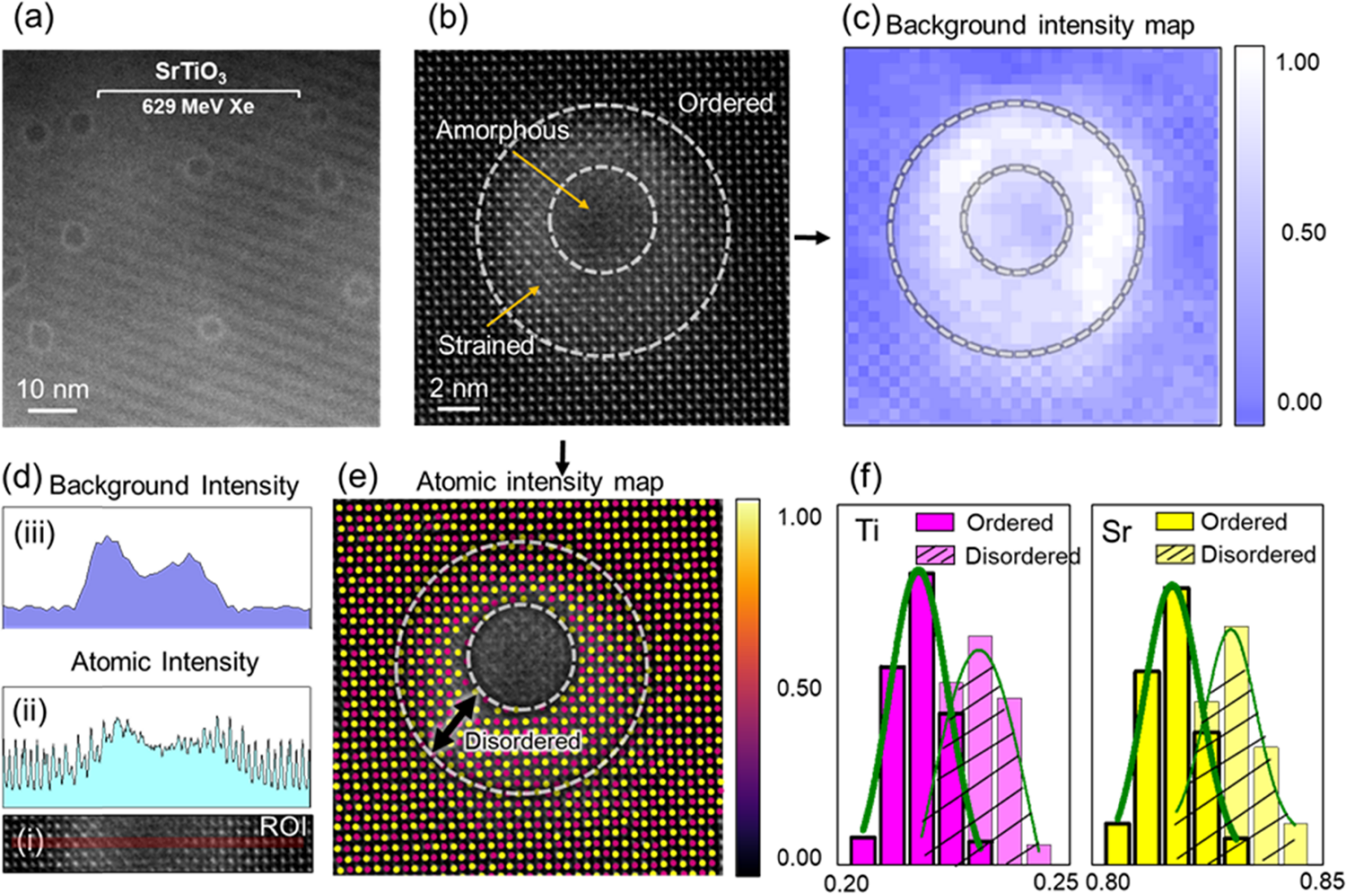
ADF image of ion tracks in SrTiO_3_ in a plan-view sample. (b) Atomic-resolution ADF image of a single ion track with the corresponding (c, d) background intensity map and (e) atomic intensity map. (f) Distribution of Sr and Ti cation intensities in ordered and disordered crystalline regions.

The effect of disorder is more pronounced in the background (oxygen) intensity than in the cationic columns (Figure 6d), suggesting that oxygen anions undergo larger displacements from their ideal lattice sites, while the cation sublattice remains comparatively intact or undergoes partial recrystallization postirradiation. Additionally, atom-by-atom identification of Sr and Ti based on column intensities shows a broader distribution of intensities in the disordered regions relative to the ordered matrix, further confirming structural deviation, as illustrated in Figure 6e,f.

An additional noteworthy observation is the markedly different response of ion tracks in Sr­(HE)­O_3_ and SrTiO_3_ to electron beam (e-beam) irradiation. As demonstrated in Figure 7, ion tracks in Sr­(HE)­O_3_ remain structurally stable under prolonged e-beam exposure, whereas those in SrTiO_3_ exhibit rapid recrystallization. The stability of the ion tracks in Sr­(HE)­O_3_ remains consistent independent of the track morphology, as shown in the figure. The series of images shows the evolution of ion tracks under electron irradiation after 0, 1.5, 3, and 5 s.

**Figure 7:**
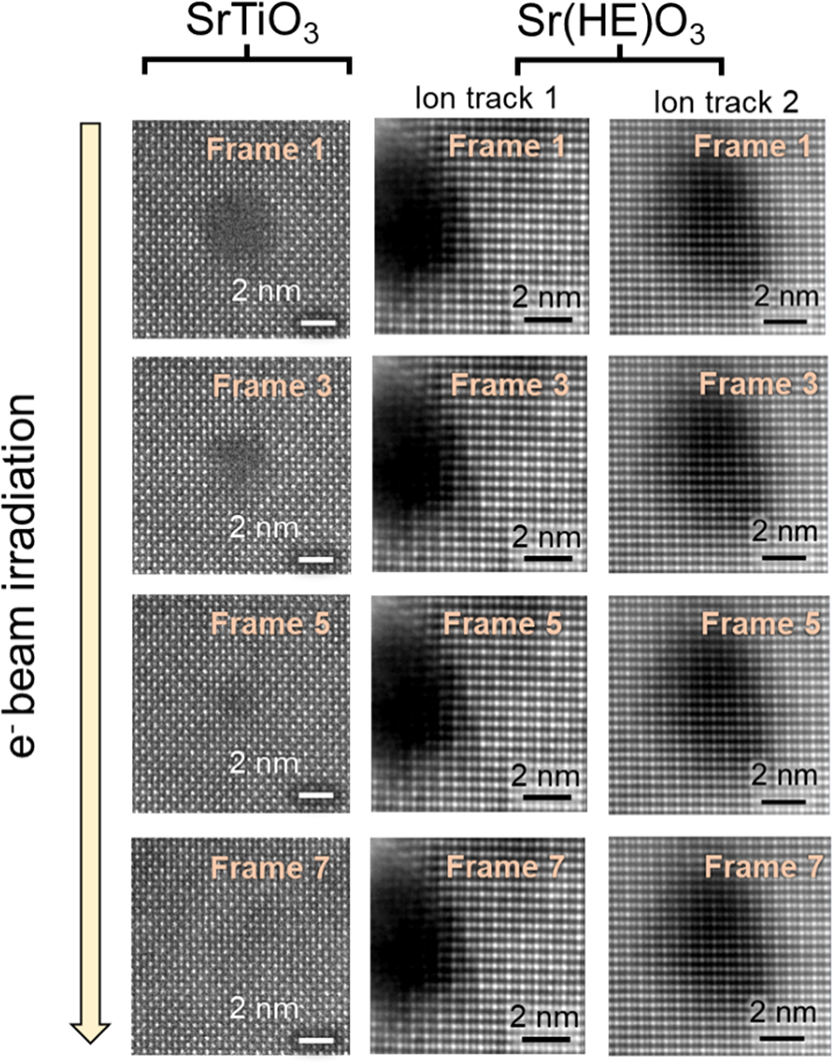
ADF image tracks in SrTiO_3_ and Sr­(HE)­O_3_ for various electron beam exposure times. Electron beam exposure results are presented for two ion tracks in Sr­(HE)­O_3_ having amorphous and disordered crystalline morphologies. The investigations were conducted in STEM with a 200 keV electron beam.

In Sr­(HE)­O_3_, atomic column intensity maps confirm that the positions of both the A-site (Sr) and B-site (high-entropy) cations remain unchanged, reflecting a high degree of structural stability under electron irradiation. In contrast, the amorphous ion tracks in SrTiO_3_ gradually recrystallize into the perovskite phase. The recrystallization begins at the interface between the disordered core and the surrounding crystalline matrix and progresses radially inward.^51^ This transformation is facilitated by local transient heating and annealing effects under the electron beam, enabling the rearrangement of the Sr and Ti cation sublattices at room temperature with substantially less energy than that required for ion beam-induced crystallization.

Electron beam-induced crystallization in SrTiO_3_ is driven by multiple mechanisms, including enhanced defect mobility from elastic and inelastic energy transfer,^52,53^ ionization-induced bond rearrangements,^38,54^ and localized beam heating.^55^ The electronic energy loss from SHIs (∼28.5 keV/nm) can excite electrons and induce sufficient local atomic perturbations to promote bond rearrangements, vacancy-interstitial recombination, and defect migration. The relatively low threshold for recrystallization in amorphous SrTiO_3_ is also attributed to the presence of thermodynamically unstable cation–oxygen bonds, which are susceptible to rearrangement under e-beam exposure. This process involves localized atomic hopping and rotation of TiO_6_ polyhedra at the amorphous–crystalline interface.

In contrast, Sr­(HE)­O_3_ demonstrates remarkable resistance to e-beam-induced recrystallization. This resilience is attributed to the chemical complexity at the B-site, composed of multiple cations (Zr, Sn, Ti, Hf, and Nb), which leads to a broad distribution of B–O polyhedra and associated lattice distortion. The resulting sluggish atomic diffusion, analogous to the diffusion barriers observed in metallic high-entropy alloys, impedes the atomic rearrangements necessary for recrystallization. The size mismatch and lattice frustration among cations with dissimilar ionic radii elevate the energy barriers for defect migration, interstitial movement, and grain growth, thereby enhancing the radiation tolerance.

Moreover, charge disorder resulting from the inclusion of cations with varying valence states (+4 for Hf, Ti, Sn, and Zr; +5 for Nb) further suppresses recrystallization. Such chemical and charge complexity increases the threshold energy required for ordering, making the system more resistant to beam-induced structural transitions. This behavior also explains the formation of discontinuous ion tracks with reduced diameters in Sr­(HE)­O_3_ under SHI irradiation. In contrast to SrTiO_3_, the ionization energy imparted during e-beam exposure is insufficient to overcome the kinetic barriers to ordering within the amorphous matrix of Sr­(HE)­O_3_, which contains chaotically arranged polyhedra containing chemically diverse elements.

The phase change or recrystallization under irradiation, particularly under electron irradiation, can have different implications, depending on the context. Structural stability is crucial for ensuring a reliable interpretation of intrinsic material properties, especially for functional applications that represent emerging research directions in HEOs. In such cases, maintaining a stable crystalline framework under irradiation is generally desirable as it reflects the robustness of the material against defect accumulation or structural degradation.

Conversely, in systems where the structure–property relationships are well-understood, controlled recrystallization can be advantageous for tailoring phase formation and optimizing performance. Such controlled structural reorganization may be beneficial for certain functionalities recently explored in HEOs, including ionic transport, quantum information sciences, catalysis, ferromagnetism, and electronic applications.^56–59^ Therefore, the stability or recrystallization behavior under irradiation should be interpreted in the context of the desired application, either as a measure of structural resilience or as a pathway for tunable property enhancement.

## Conclusions

This study reveals that the irradiation of SrTiO_3_ and Sr­(HE)­O_3_ with 629 and 744 MeV Xe ions leads to the formation of discontinuous ion tracks with distinct structural characteristics. Atomic-resolution ADF-STEM imaging shows that ion tracks in Sr­(HE)­O_3_ have a much reduced diameter (∼5 ± 2 nm) than those in SrTiO_3_ (∼8.5 ± 1 nm), despite the greater structural complexity of Sr­(HE)­O_3_. While SrTiO_3_ tracks display amorphous cores and strain fields at the amorphous–crystalline interface, such strain features are absent in Sr­(HE)­O_3_. According to the inelastic thermal spike model, the smaller track radii in Sr­(HE)­O_3_ can be ascribed to the lower thermal conductivity and slightly larger electron–phonon mean free path, which both confine the thermal spike and local energy deposition. STEM-EDS analysis confirms compositional homogeneity within the tracks, while STEM-EELS reveals distortions of the Ti–O octahedra in both materials, leading to peak broadening and shifts in Ti L_2,3_ and O K edges. Notably, ion tracks in Sr­(HE)­O_3_ remain structurally stable under electron beam exposure, in contrast to the rapid recrystallization observed in SrTiO_3_. This stability is attributed to the presence of multiple cations with different ionic radii and valence states in Sr­(HE)­O_3_, which suppress atomic diffusion and raise the recrystallization threshold by introducing lattice and charge disorder. Together, these findings underscore the enhanced irradiation tolerance of Sr­(HE)­O_3_, governed by its compositional complexity, and highlight its potential as a model system for studying defect dynamics and energy dissipation in complex oxides.

## Methods

### Materials Synthesis

The stoichiometric Sr­(HE)­O_3_ (HE: Zr_0.2_Sn_0.2_Ti_0.2_Hf_0.2_Nb_0.2_) perovskite used in this study was synthesized using a conventional solid-state reaction approach.^60^ Individual starting metal oxides were dried overnight at 150 °C to remove moisture. Then, a stoichiometric mixture of oxide powders was ball-milled in a zirconia canister for 1 h. The powder was then pressed into 0.5 in.-diameter pellets that were heated at 5 °C/min to a reaction temperature of 1500 °C, held for 10 h, and then furnace-cooled. X-ray diffraction analysis confirmed the structure as a single-phase cubic (*Pm* $\overset{-}{3}$ *m*) perovskite. Single crystals of SrTiO_3_ were commercially purchased.

### Ion Irradiation

SHI irradiation of single crystalline SrTiO_3_ and a polycrystalline sample of Sr­(HE)­O_3_ was performed with 629 and 774 MeV Xe ions, respectively. The irradiation of SrTiO_3_ was conducted to a fluence of 1 × 10^11^ ions/cm^2^ by using the UNILAC linear accelerator at the GSI Helmholtz Center for Heavy Ion Research in Darmstadt (Germany). The irradiation of Sr­(HE)­O_3_ was conducted to a fluence of 1 × 10^12^ ions/cm^2^ in the terminal chamber of the Separated Sector Cyclotron (SSC) at the National Laboratory of Heavy Ion Research Facilities in Lanzhou (HIRFL) (China). According to the SRIM-13 code, the ranges of 629 MeV Xe ions in SrTiO_3_ and 774 MeV Xe ions in Sr­(HE)­O_3_ are ∼30 and ∼35 μm, respectively. For the track analysis at ∼5 μm ion penetration depth, the corresponding energy losses for both beams are very similar (∼28.5 keV/nm) (Figure 1a).

### STEM Characterization

HAADF imaging for the plan-view and cross-sectional views was conducted in a fifth-order aberration-corrected scanning transmission electron microscope (STEM) (Nion UltraSTEM200), operating at 200 keV. The plan-view and cross-sectional samples were prepared by conventional mechanical polishing followed by ion milling. Mechanical polishing was done using diamond lapping films (15 to 1 μm) in the Allied Multiprep polishing system to thin down the samples down to ∼12 μm thickness. Subsequently, ion milling was done in a Gatan PIPS-II instrument to make electron-transparent samples for the STEM studies. HAADF imaging was performed using an inner semiangle of 65 mrad. We observed considerable strain contrast in the disordered region. Thus, the images will be referred to as annular dark field (ADF) in the entire study. ABF imaging illustrating oxygen atom positions in SrTiO_3_ was done with collection of semiangles between 15 and 30 mrad. The electron probe current used in the experiment was 28 ± 2 pA. Electron energy loss spectroscopy (EELS) data were acquired with a collection angle of 48 mrad. An energy dispersion of 0.1 eV/channel and a spatial pixel size of 0.04 nm were utilized to acquire fine-structure EELS data over the selected area of interest. To examine the elemental distribution, the specimens were analyzed in an aberration-corrected 200 keV JEOL-NEOARM microscope equipped with an energy-dispersive spectroscopy (EDS) detector.

### STEM Image Data Analysis

The atomic-resolution ADF images were analyzed by extracting and separating the intensities of atomic columns of Sr and Ti and the background. In the ADF images obtained in the [001] zone axis, atomic columns purely contain only Sr or Ti atoms and can be distinguished by their intensity, as shown in Figure 1. This identification of cations generates information on atomic column coordinates and is mapped in terms of the localized strain, which is relevant to understanding the cation ordering/disordering. The background intensity between the cationic atomic columns consists of background noise from the detector, and the intensity variations appear due to the strain contrast of the O anions. The mapping of this background intensity provides qualitative information on the ordering/disordering of oxygen column positions that can vary due to octahedral tilting or distortions subsequent to the irradiation.^32^ The identification of atomic columns in the atomic-resolution ADF images was performed using deep convolutional neural networks (DCNNs) where a DCNN is trained with either a Tensor Processing Unit (TPUv2) or with a Tesla K80 Graphical Processing Unit (GPU) in Google Colaboratory with Tensorflow and Keras deep learning libraries and subsequently applied to analyze the acquired imaging data. Positions/coordinates of atoms (Sr and Ti) in the ADF images were identified using AtomAI open-source software, a Python library based on the Pytorch deep learning frameworks.^61,62^ In this framework, the positions of Sr and Ti atomic columns are identified using the mean prediction model^63^ that helps ascertain the atomic displacements, lattice distortions, and localized strain at the atomic level.

### Inelastic Thermal Spike (iTS) Model Calculations

The iTS model^64^ was used to calculate the energy deposition in SrTiO_3_ and Sr­(HE)­O_3_ due to 629 and 774 MeV Xe ions, respectively. The iTS describes the transfer and dissipation of the energy of a fast-moving ion to the electronic and after electron–phonon coupling to the atomic subsystems. The model is based on two coupled heat diffusion equations, one describing the temperature evolution of the electronic subsystem (eq 1) and the other describing the temperature evolution of the atomic subsystem (eq 2):

$$C_{e}(T_{e})\frac{\partial T_{e}}{\partial t}=\frac{1}{r}\frac{\partial }{\partial r}\left[rK_{e}(T_{e})\frac{\partial T_{e}}{\partial r}\right]-g(T_{e}-T_{a})+A(r,t)$$ 1

$$C_{a}(T_{a})\frac{\partial T_{a}}{\partial t}=\frac{1}{r}\frac{\partial }{\partial r}\left[rK_{a}(T_{a})\frac{\partial T_{a}}{\partial r}\right]+g(T_{e}-T_{a})$$ 2

Here, *C*_e_ and *C*_a_ are the heat capacities of the electronic and atomic systems, *K*_e_ and *K*_a_ are the electronic and lattice thermal conductivities, respectively, and *g* is the electron–phonon (e-ph) coupling parameter. The term *A*(*r*, *t*) describes the spatial and temporal energy deposition from the incident ion to the electrons.^65^ The electronic stopping used in the calculations was obtained by using the SRIM code. For both systems, *C*_e_ is 1 J cm^–3^ K^–1^ and *K*_e_ is equal to *C*_e_ *D*_e_, where *D*_e_ is the thermal diffusivity and has a value of 1.0 cm^2^ s^–1^.^64,66^ The e-ph coupling parameter *g* is equal to *K*_e_/λ^2^ where λ is the e-ph mean free path. Based on the energy gap of SrTiO_3_, λ = 4.7 nm. We measured the energy gap of Sr­(HE)­O_3_ to be 3.25 eV, which is the same as that for SrTiO_3_. The e-ph mean free path λ = 1/(ρ_n_σ), with ρ_n_ being the number density and σ the scattering cross section per atom (the area of the thermally fluctuating atom with which the passing electron interacts with). The number density ρ_n_ is equal to (*N*_A_ρ)/Α, where *N*_A_ is the Avogadro number, ρ is the atomic density, and *A* is the mean atomic mass. The density of Sr­(HE)­O_3_ is a little higher than that of SrTiO_3_ (5.77 and 5.12 g/cm^3^, respectively), and *A*(Sr­(HE)­O_3_) = 48.3 and *A*(SrTiO_3_) = 36.7. With the same σ for both, the e-ph mean free path is expected to be smaller for Sr­(HE)­O_3_. Additionally, it has been shown that HEOs have enhanced electrical conductivity,^67^ which means that the electronic diffusivity is also expected to be enhanced, which further supports that λ is larger for Sr­(HE)­O_3_ based on the relationship λ = *D*_e_ *C*_e_. Since the value of λ for Sr­(HE)­O_3_ is not known, we use a 25% larger value than the one for SrTiO_3_. For SrTiO_3_, *K*_a_ is 11.2 W m^–1^ K^–1^ at 300 K and *C*_a_ is 0.544 J cm^–3^ K^–1^. Experimental studies have shown that the conductivity of HEOs is significantly lower than the one in their traditional counterparts; hence, here, we use a *K*_a_ of 2 W m^–1^ K^–1^ ^68,69^ since the conductivity is not known. Experiments on HEOs have shown that the specific heat in HEOs is significantly reduced, so we use *C*_a_ that is 50% smaller for Sr­(HE)­O_3_.^70^

## Supplementary Material



## Notes

Additional discussion on the tolerance factor of high-entropy oxides (Table S1); scanning transmission electron microscopy data: EDS line scan (Figure S1), EELS (Figure S2), and ADF and ABF images (Figure S3); statistical analysis of atomic-resolution images in Figures 5 and 6 of the manuscript (Table S2) (PDF)
