# Supplementary material for: Unveiling Swift Heavy Ion Track Morphology in Sr-Based High-Entropy Perovskites
Source: ACS Nano. 2026 Jan 10;20(8):6632–43. doi: 10.1021/acsnano.5c13654 (PMC12961946; doi:10.1021/acsnano.5c13654)
Supplement: Supplementary file 1 [file nn5c13654_si_001.pdf]

Supplemental information

Unveiling Swift Heavy Ion Track Morphology in Sr-Based High-Entropy Perovskites

Ashish Kumar Gupta[1], Eva Zarkadoula[2], Brianna L. Musico[3], Jordan Hachtel[2], Manuel A. Roldan[4], Vikas Reddy Paduri[1], Colby Harris[1], Ramji Subedi[1], Maxim Ziatdinov[5], Sergei V. Kalinin[6], Christina Trautmann[7], Veerle Keppens[6], Jie Liu[8], Yanwen Zhang[9], William Weber[6*], and Ritesh Sachan[1*]

[1] School of Mechanical and Aerospace Engineering, Oklahoma State University, Stillwater, OK 74078, USA
[2] Center for Nanophase Materials Sciences, Oak Ridge National Laboratory, Oak Ridge, Tennessee 37831, USA
[3] Sigma Division, Los Alamos National Laboratory, Los Alamos, NM, 87545, USA
[4] Eyring Materials Center, Arizona State University, Tempe, Arizona 85287, USA
[5] Physical Sciences Division, Pacific Northwest National Laboratory, Richland, Washington, 99352, USA
[6] Department of Materials Science and Engineering, University of Tennessee, Knoxville, Tennessee 37996, USA
[7]GSI Helmholtzzentrum für Schwerionenforschung, Darmstadt, 64291, Germany
[8] Institute of Modern Physics, Chinese Academy of Sciences, Lanzhou, 730000, China
[9]Department of Mechanical and Materials Engineering, Queen's University, Kingston, Ontario, K7L3N6, Canada

*Corresponding author. e-mail: rsachan@okstate.edu, wjweber@utk.edu

Global cubic stability: Using Shannon radii (for A site: Sr^{2+}, 12-coord: 1.44 Å; X site: O^{2-}: 1.40 Å; B-site, 6-coord: Ti^{4+} 0.605 Å, Nb^{5+} 0.64 Å, Sn^{4+} 0.69 Å, Zr^{4+} 0.72 Å, Hf^{4+} 0.71 Å) and equiatomic B-site averaging, we obtain r_B=0.673 used to measure a Goldschmidt's tolerance factor.

Goldschmidt Tolerance factor:

$$t = \frac{r_A + r_X}{\sqrt{2}(r_B + r_X)}$$

Which gives us a global cubic stability factor of t = 0.969 < 1,

Octahedral factor, $\mu = r_B/r_O = 0.481$

These lie within standard perovskite windows, supporting overall cubic stability.

Table S1:

	Value	Notes
Sr^{2+} radius, r_A [Å]	1.44	Shannon, CN=12
O^{2-} radius, r_O [Å]	1.40	Conventional for ABO_3
Mean B-site radius r_B [Å]	0.673	Comp.-weighted (CN=6)
Tolerance factor t	0.969	Goldschmidt
Octahedral factor μ	0.481	$\mu = r_B / r_O$
B-site mismatch δ_B [%]	6.504	Size-variance metric

Local size mismatch ("local tolerance"): We quantify B-site size disorder via the radius-variance metric, Δr_B and construct a **local tolerance factor** distribution t_{local} by

$$\Delta t = -\frac{t}{(r_B + r_O)} \Delta r_B$$

For $Sr(ZrNbSnTiHf)O_3$ we find average $t_{local} \approx 0.969$, with a spread of $\sigma_t \approx 0.021$ implying a local strain of 2.1%. This spread of t_{local} imply local B–O bond-length variations and elevated elastic energy at the octahedral scale.

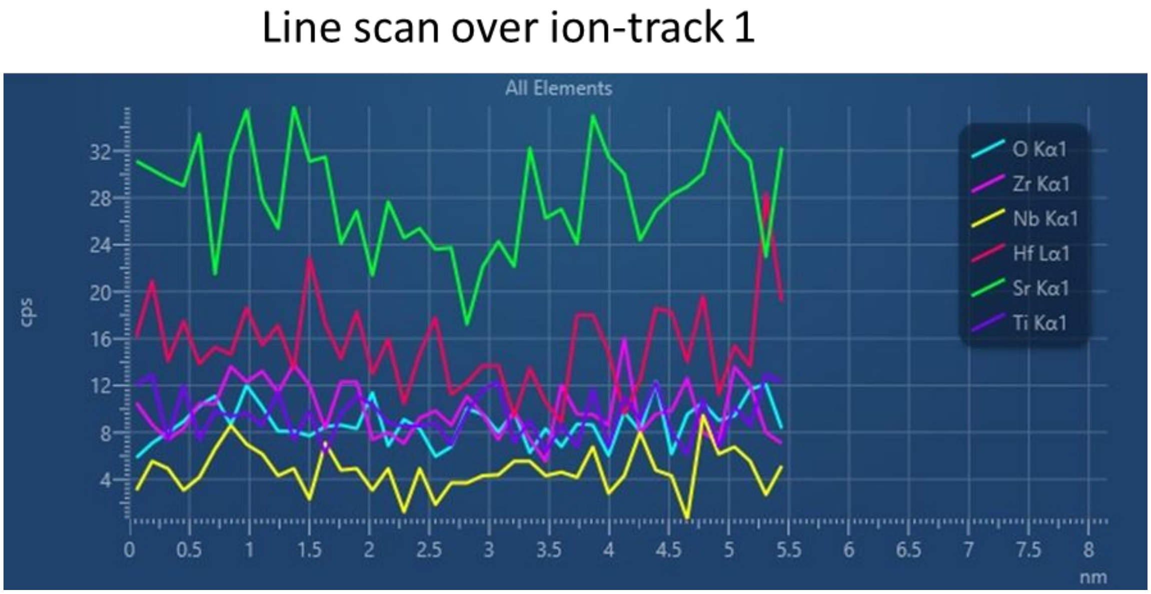

Figure S1. Line scan over ion-track shown in Figure 3.

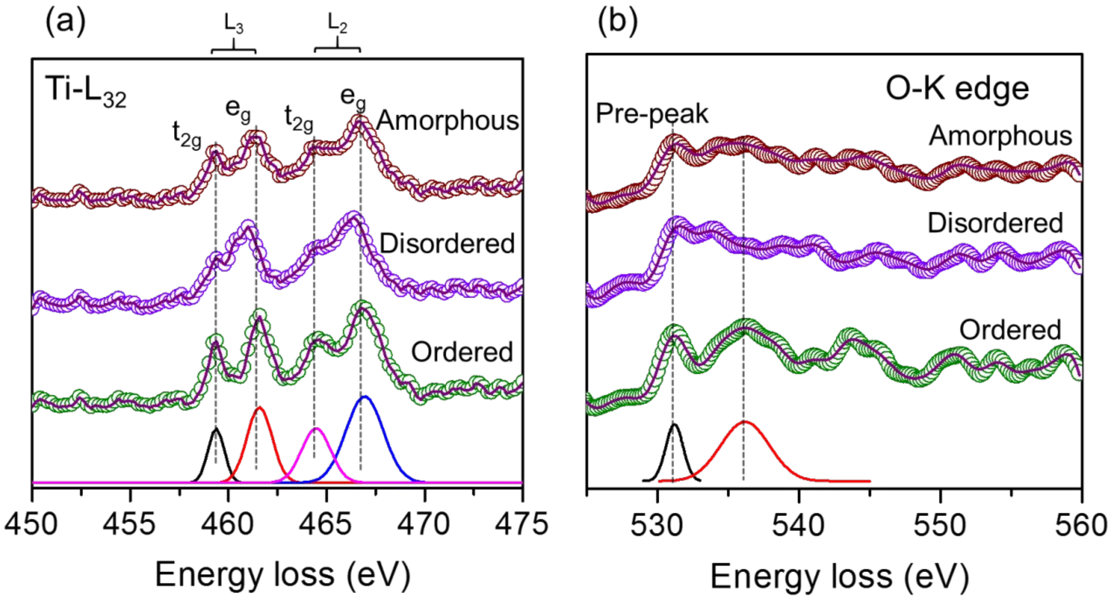

Figure S2. (a) Energy-loss near-edge structure spectra of Ti-L_{32} edge (456 eV), and (b) the O-K edge (532 eV) spectra from the ordered, disordered, and amorphous regions, respectively.

Table S2: Statistical analysis, intensity mean and FWHM (in parentheses) in ordered and disordered regions of ion-tracks in $SrTiO_3$ and $Sr(HE)O_3$ relevant to Figure 5 and 6 of manuscript.

Material	Atomic Column	Ordered intensity mean (FWHM)	Disordered intensity mean (FWHM)
$SrTiO_3$	Sr	0.8504 (0.0253)	0.8304 (0.0257)
	Ti	0.4604 (0.0232)	0.4402 (0.0255)
$Sr(HE)O_3$	Sr	0.8500 (0.0229)	0.8300 (0.0235)
	HE	0.4600 (0.0200)	0.4400 (0.0224)

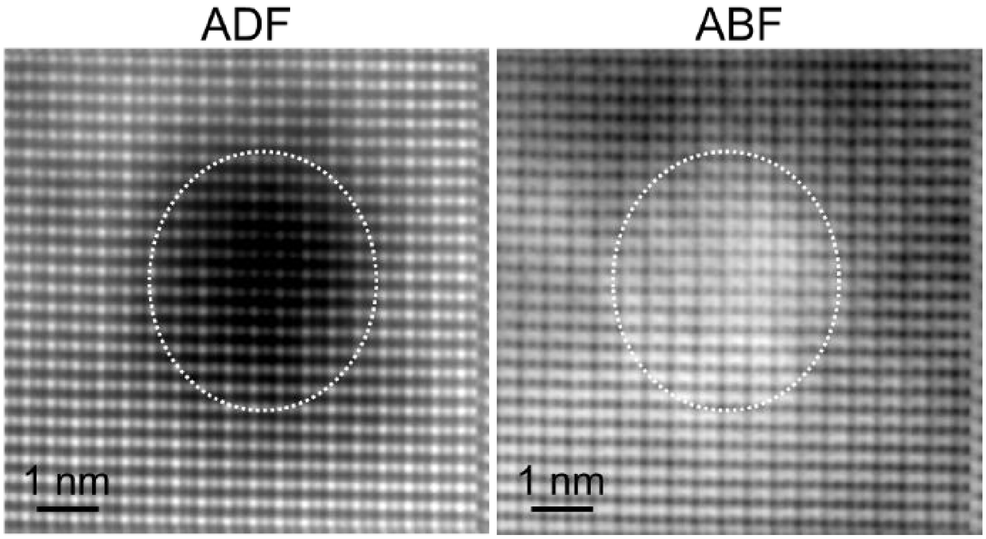

Figure S3. (a) ADF and (b) ABF image of disordered ion-track.
